# The microglial "activation" continuum: from innate to adaptive responses

**DOI:** 10.1186/1742-2094-2-24

**Published:** 2005-10-31

**Authors:** Terrence Town, Veljko Nikolic, Jun Tan

**Affiliations:** 1 Section of Immunobiology, Yale University School of Medicine, 300 Cedar St., New Haven, CT 06520-8011, USA; 2 Neuroimmunology Laboratory, Silver Child Development Center, Department of Psychiatry and Behavioral Medicine, University of South Florida, 3515 E. Fletcher Ave., Tampa, FL 33613, USA

**Keywords:** brain, microglia, innate immunity, adaptive immunity, Toll-like receptor, inflammation, encephalitis, myelin, amyloid, vaccine, immunotherapy

## Abstract

Microglia are innate immune cells of myeloid origin that take up residence in the central nervous system (CNS) during embryogenesis. While classically regarded as macrophage-like cells, it is becoming increasingly clear that reactive microglia play more diverse roles in the CNS. Microglial "activation" is often used to refer to a single phenotype; however, in this review we consider that a continuum of microglial activation exists, with phagocytic response (innate activation) at one end and antigen presenting cell function (adaptive activation) at the other. Where activated microglia fall in this spectrum seems to be highly dependent on the type of stimulation provided. We begin by addressing the classical roles of peripheral innate immune cells including macrophages and dendritic cells, which seem to define the edges of this continuum. We then discuss various types of microglial stimulation, including Toll-like receptor engagement by pathogen-associated molecular patterns, microglial challenge with myelin epitopes or Alzheimer's β-amyloid in the presence or absence of CD40L co-stimulation, and Alzheimer disease "immunotherapy". Based on the wide spectrum of stimulus-specific microglial responses, we interpret these cells as immune cells that demonstrate remarkable plasticity following activation. This interpretation has relevance for neurodegenerative/neuroinflammatory diseases where reactive microglia play an etiological role; in particular viral/bacterial encephalitis, multiple sclerosis and Alzheimer disease.

## Introduction

Microglia are somewhat enigmatic central nervous system (CNS) cells that have been traditionally regarded as CNS macrophages (MΦs). They represent about 10% on average of the adult CNS cell population [1]. In mice, microglial progenitors can be detected in neural folds at the early stages of embryogenesis. Murine microglia are thought to originate from the yolk sac at a time in embryogenesis when monocyte/Mφ progenitors (of hematopoeitic origin) are also found [1,2]. Based on this observation, it is now generally accepted that adult mouse microglia originate from monocyte/MΦ precursor cells migrating from the yolk sac into the developing CNS. Once CNS residents, these newly migratory cells actively proliferate during development, thereby giving rise to the resident CNS microglial cell pool. More recently however, it has been shown that bone marrow-derived cells can enter the CNS and become cells that phenotypically resemble microglia in the adult mouse [3-5]. Interestingly, under conditions of CNS damage such as stroke, cholinergic fiber degeneration, or motor neuron injury, Priller and colleagues found that green fluorescent protein-labeled bone marrow cells could enter the CNS and take up a microglial phenotype [6].

Microglia normally exist in a quiescent (resting) state in the healthy CNS, and are morphologically characterized by a small soma and ramified processes. However, upon "activation" in response to invading viruses or bacteria or CNS injury, microglia undergo morphological changes including shortening of cellular processes and enlargement of their soma (sometimes referred to as an "amoeboid" phenotype). Activated microglia also up-regulate a myriad of cell surface activation antigens and produce innate cytokines and chemokines (discussed in detail below). As the microglial lineage originates from peripheral myeloid precursor cells, it is helpful to consider the activation states of such peripheral innate immune cells to better understand the nature of microglial activation.

### Classical roles of peripheral innate immune cells

It is now widely accepted that both innate and adaptive arms of the immune system play important roles in maintaining immune homeostasis. However, little attention was paid to the evolutionarily much older innate immune system until the late Charlie Janeway proposed the involvement of innate mechanisms in vertebrate immunity. Specifically, Janeway pioneered the idea that lymphocyte activation could be critically regulated by the evolutionarily ancient system of antigen clearance by phagocytic cells of myeloid origin. Together with Ruslan Mezhitov, he originated the concept that these phagocytic innate immune cells recognize pathogen-associated molecular patterns (PAMPs) through pattern recognition receptors, the most notable examples being a set of phylogenetically conserved, germ-line encoded Toll-like receptors (TLRs, currently 11–12 members, [7-10]), resulting in expression of cell-surface activation molecules [for example, major histocompatibility complex (MHC) class I and II, B7.1, B7.2, and CD40] and secretion of innate cytokines [i.e., tumor necrosis factor α (TNF-α), interleukin (IL)-1, IL-6, IL-12, and IL-18] [11,12]. Once activated, the innate arm of the immune response calls adaptive immune cells into action, and both branches act in concert to promote neutralization and clearance of invading pathogens. Thus, innate immune cells are able to discriminate "non-infectious self" from "infectious non-self" and thereby form the first line of defense against invading bacteria and viruses (for reviews see [13-15]).

#### The macrophage: prototypical phagocyte

MΦs are quintessential phagocytes whose primary role is to engulf pathogens such as invading bacteria and to remove debris and detritus, i.e., remnants of apoptotic cells. Tissue MΦs develop when blood monocytes enter into the various organs and tissues and differentiate into specialized, site-specific MΦs depending on their anatomical location, such as alveolar MΦs (lung), histiocytes (connective tissue), kupffer cells (liver), mesangial cells (kidney), osteoclasts (bone), or microglia (brain) [16]. Resting MΦs are both weak phagocytes and weak lymphocyte activators [17]. Upon activation however, for example in response to TLR stimulation by PAMPs, their phagocytic potential greatly enhances [18] and they up-regulate cell-surface co-stimulatory molecules and produce pro-inflammatory innate cytokines as mentioned above. Typically, engulfment of the pathogen by phagocytosis triggers a "respiratory burst" involving production of reactive oxygen species such as superoxide and peroxinitrite that kill the pathogen [17,19]. In addition, activated MΦs up-regulate cell-surface Fc receptors that aid in phagocytosis of pathogens opsonized by antibodies produced by plasma cells [20,21]. On the other hand, in response to debris from apoptotic cells, the MΦ mounts a phagocytic response essentially in the absence of pro-inflammatory cytokines [22]. The most likely reason for this anti-inflammatory phagocytic response is that pro-inflammatory cytokines such as TNF-α promote bystander injury which may further damage tissues in which the apoptotic cells reside. Thus, MΦs are highly capable of "innate activation" characterized by a strong phagocytic response sometimes accompanied by pro-inflammatory cytokine production (for a review see [23]).

#### The dendritic cell: professional antigen presenting cell

Whereas MΦs have limited ability to process and present antigen to T cells, dendritic cells (DCs) are considered professional antigen presenting cells (APCs). DCs can be found under the epithelia and in most organs where they capture and process non-self antigens, migrate to lymphoid organs, and present antigen in the context of MHC to CD4+ and CD8+ T lymphocytes. With their many finger-like cellular processes, DCs are morphologically optimized to simultaneously display antigen to many T cells. Like MΦs, DCs respond to invading pathogens by recognizing PAMPs through TLRs, and subsequently phagocytose and process antigen. DCs then up-regulate cell-surface co-stimulatory molecules and secrete innate cytokines and chemokines (typically at levels an order of magnitude higher than those secreted by MΦs) to promote recruitment and activation of CD4+ and/or CD8+ T lymphocytes. There are three generally accepted classifications of DCs in mice: plasmacytoid (p) DCs (CD11c^lo^, CD11b^lo^, B220+, CD8-), lymphoid (l) DCs (CD11c^+^, CD11b-, CD8+), and myeloid (m) DCs (CD11c+, CD11b+, B220-, CD8-, there are several subtypes, [24]). In humans, there are clearly two distinct subsets of DCs: pDCs (CD11c-, CD11b-, CD14-, CD45RA+) and monocyte DCs (CD11c+, CD11b+, CD14+, CD45RA-) (for a review see [25]). DC classes differ from each other predominately in tissue distribution, production of specific cytokines, TLR expression, and ability to promote innate *versus *adaptive immune responses (for a review see [15]). For example, freshly isolated human pDCs express TLR7 and 9, whereas mDCs express TLR1, 2, 3, 5, 6, and 8 [26-28]. Stimulation of human pDCs or monocytic DCs with synthetic TLR7 ligands induces the secretion of interferon (IFN)-α (important for anti-viral innate immunity) or IL-12 [a key inducer of the adaptive T helper (Th) type I response], respectively [29]. Similarly, stimulation of TLR9 via DNA containing unmethylated CpG motifs results in IFN-α secretion by pDCs and IL-12 production by murine mDCs [30]. Despite these relative differences between DC classes, the major role of DCs on the whole remains; they act as potent APCs capable of strongly activating T lymphocytes. Their APC capacity is much stronger than that of MΦs, as DCs are able to directly activate naïve T cells whereas MΦs are not [15]. Thus, by virtue of their ability to promote T cell activation responses, DCs are highly capable of "adaptive activation". Activation markers of phagocytosis and APC responses in various innate immune cells are presented in Table 1.

**Table 1:** Phagocytic and antigen presenting cell responses of immune cells. Note: ND, assay not performed; +/-, weak response; +, modest response; ++, strong response

**Surface marker**	**Phagocytosis**	**APC function**	**References**
CD36	++	+	[91] [92] [93]
CD40	ND	++	[58] [94] [95]
CD80	ND	++	[94] [95] [96]
CD86	ND	++	[94] [95] [96]
MHC II	ND	++	[94]
CD11c	ND	++	[58]
CD40L	+/-	++	[58] [80]
**Cytokine**			
IL-1β	ND	++	[97]
IL-6	ND	++	[98]
IL-12	ND	++	[98] [97]
TNF-α	+	++	[98] [80] [97]
IFN-γ	-/+	++	[80]
IL-4	++	-/+	[80]
IL-10	++	-/+	[80]
TGF-β1	++	ND	[99]

## Microglial activation after toll-like receptor stimulation: a mixed response

Recent evidence indicates that microglia, like their peripheral innate immune cell counterparts, express a wide array of TLRs, including mRNA for TLRs 1–9 in mice [31] and in humans [32]. Furthermore, many of these TLRs have been shown to be functional, allowing microglial recognition of a variety of PAMPs. For example, Kielian and coworkers found that heat-killed Staphylococcus aureus and its cell wall product peptidoglycan (PGN) are able to stimulate innate activation of microglia characterized by pro-inflammatory cytokine and chemokine production [33]. Those authors found that the effect of PGN was critically dependent on TLR2, as TLR2-deficient mice demonstrated reduced cytokine and chemokine production after PGN challenge [34]. Furthermore, murine microglia respond to the TLR9 agonist, unmethylated CpG-DNA, by secreting numerous pro-inflammatory innate cytokines (probably responsible for neurotoxicity in oganotypic brain slice cultures treated with CpG-DNA [35]), by up-regulating co-stimulatory cell surface molecules, and by promoting adaptive activation by secreting IL-12 to affect T cell activation [36]. In two recent studies, murine microglial pro-inflammatory responses to bacterial lipopolysaccharide (LPS), a known TLR4 ligand, resulted in dramatic injury to cultured oligodendrocytes [37] and neurons [38], further demonstrating microglial bystander injury after TLR stimulation (probably mediated by over-production of innate pro-inflammatory cytokines). It has recently been shown that microglia respond to poly I:C [a synthetic double-stranded (ds) RNA analog thought to be recognized by TLR3, [39]] by producing pro-inflammatory cytokines and chemokines [40], and microglial pro-inflammatory responses to dsRNA seem to be dependent on TLR3, as TLR3-deficient microglia have blunted innate cytokine responses *in vitro *and markedly reduced cell surface activation markers in brain after poly I:C stimulation (Town *et al*., submitted). Finally, infection with West Nile virus, a retrovirus that produces dsRNA during its life cycle, results in profound microglial activation as assessed by pro-inflammatory cytokine production *in vitro *and cell surface activation markers *in vivo*, effects that are dramatically reduced in TLR3-deficient animals [41].

**Figure 1:**
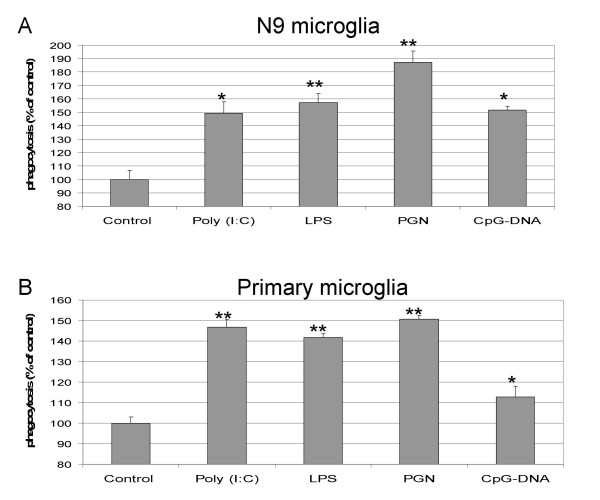
PAMP stimulation results in enhanced microglial phagocytosis. N9 cells (A) or primary cultured microglia from C57BL/6 mice (B) were pre-stimulated with the PAMPs indicated (Poly I:C, 50 μg/mL; LPS, 50 ng/mL; PGN, 50 μg/mL; CpG-DNA, 1 μM) for 6 hours. Cells were rinsed in complete RPMI 1640 media (containing 5% fetal calf serum and 1 mM penicillin/streptomycin) and then cultured for an additional 1 hour at 37°C or at 4°C (control) with yellow-green fluorescent latex beads (1:1000, Sigma). After extensive rinsing, microglia were subjected to fluorescence-activated cell sorter analysis, and mean fluorescence intensity of cells cultured at 4°C was subtracted from values from cells cultured at 37°C. These figures were then normalized to untreated control microglia to obtain percentage of phagocytosis increase over baseline. Unpaired t-test was used to assess statistical significance for each treatment condition compared to control, with n = 3 wells for each condition presented; ** p < 0.001, * p < 0.05. Abbreviations used: Poly (I:C), polyinosinic : polycytidylic acid; LPS, lipopolysaccharide; PGN, peptidoglycan; CpG-DNA, unmetylated DNA containing CpG motifs.

In addition to production of pro-inflammatory cytokines and up-regulation of cell surface activation antigens, phagocytosis is a hallmark indicator of innate immune cell activation. We have recently investigated microglial phagocytosis in response to PAMP stimulation using both the N9 microglial cell line and primary cultured microglia derived from neonatal C57BL/6 mice (for methods see [42]). We pre-treated either N9 cells or primary cultured microglia with poly (I:C) (50 μg/mL), LPS (50 ng/mL), PGN (50 μg/mL), or CpG-DNA (1 μM) for 6 hours, rinsed the cells multiple times in complete RMPI 1640 media, and then cultured cells in the presence of a 1:1000 dilution of yellow-green fluorescent latex beads (Sigma) at 37°C or at 4°C (a control for non-specific incorporation of beads) for 1 hour. After this final culture period, cells were rinsed multiple times in complete media and subjected to fluorescence-activated cell sorter analysis, and mean fluorescence intensity values obtained from cells at 4°C were subtracted from figures obtained from cells cultured at 37°C. These corrected figures were then normalized for each treatment condition to values obtained from untreated control cells, yielding a percentage of phagocytosis increase over baseline. As shown in Fig. 1, both N9 and primary cultured microglia increased phagocytosis after stimulation with each PAMP studied by as much as ~190% over baseline (see PGN treatment of N9 cells, Fig. 1A).

When taking these results together with the above-mentioned reports, it seems that PAMP stimulation of TLRs produces a "mixed" microglial activation phenotype. In terms of innate responses, PAMP-stimulated microglia clearly secrete pro-inflammatory innate cytokines (i.e., TNF-α and IL-6), up-regulate cell-surface activation markers (i.e., MHC I and II, B7.1, B7.2, CD40), and increase phagocytosis. Regarding adaptive responses, particularly in the case of CpG-DNA stimulation of TLR9, reactive microglia activate T lymphocytes and may bias CD4+ T cells towards a pro-inflammatory T helper type I response by secreting IL-12 [36].

In peripheral innate immune cells, TLR responses to PAMPs seem to be dependent on at least four different TLR intracellular adapter molecules: MyD88 (involved in TLR1, 2, 4, 6, 7, 8, and 9 signaling), TRIF/TICAM-1 (mediates TLR3 and 4 signaling), TIRAP/Mal (involved in TLR1, 2, 4, and 6 responses) and TIRP/TRAM/TICAM-2 (mediates TLR4 signaling). These adapter molecules bind to the intracellular leucine-rich repeat region of the TLR and promote recruitment of additional factors such as IRAKs and TRAF6 that allow for activation of transcription factors including IRF-3 and NF-κB, which are responsible for activation of numerous innate cytokines and cell-surface activation antigen genes (for review see [43,44]). It is still unclear how different TLR responses in innate immune cells (i.e., promotion of innate *versus *adaptive responses) can be achieved when many TLRs share intracellular signaling molecules. While little work has been done on intracellular signaling following TLR stimulation in microglia, it is likely that microglia utilize the same signaling cascades described for MΦs and DCs.

## Adaptive response of activated microglia in demyelinating disease via CD40-CD40 ligand interaction

### Brain inflammation in demyelinating disease

Experimental autoimmune encephalomyelitis (EAE) is a mouse model of the human disease multiple sclerosis (MS), an autoimmune disease characterized by inflammatory CNS demyelinating lesions accompanied by motor disturbances. EAE can be induced in different strains of mice by subcutaneous or intraperioteneal inoculation with adjuvant plus epitopes found in myelin such proteolipid protein, myelin basic protein, or myelin oligodendrocyte glycoprotein. The disease is critically dependent on activation of pro-inflammatory CD4+ T helper type I (Th1) cells by APCs, and these auto-aggressive Th1 cells can be adoptively transferred to non-diseased recipient mice that subsequently develop disease. EAE is characterized by paralysis, typically beginning in the tail and hind limbs and progressing to the fore limbs. In the SJL mouse strain, animals develop a relapsing-remitting form of the disease while C57BL/6 mice manifest paralysis that progressively worsens until death. Upon histopathological analysis, brains from EAE mice generally show infiltration of Th1 cells (and other lymphocytes including MΦs and DCs) and activation of microglia, typically in white matter regions where demyelinating lesions are found (for a review see [45-47]).

### CD40-CD40 ligand interaction in experimental autoimmune encephalomyelitis

Immune/inflammatory cells receiving a primary stimulus (i.e., MHC-T cell receptor interaction between APCs and T lymphocytes, respectively) typically require co-stimulatory signals *via *other pairs of molecules in order to become activated [for instance, the B7-CD28 and/or CD40-CD40 ligand (L) dyads in APC/T-cell activation; [48]. CD40L is a key immunoregulatory molecule that plays a co-stimulatory role in the activation of immune cells from both the innate and adaptive arms of the immune system, and is typically expressed by activated CD4+ and some CD8+ T cell subsets [49]. CD40 receptor, a member of TNF and nerve growth factor super-family, is expressed on many professional and non-professional APCs, including DC's, B cells, monocytes/MΦs and microglial cells [42,50-53]. Nearly 10 years ago, activated Th cells that expressed CD40 ligand (CD40L) were found in brains of MS patients, and these cells were found in close apposition to CD40-bearing cells in active demyelinating lesions [54]. The authors determined that the CD40-expressing cells were either MΦ or microglia based on staining for acid phosphatase or CD11b.

To evaluate whether the CD40-CD40L interaction was pathogenic in EAE, Gerritse and co-workers administered a CD40L neutralizing antibody to SJL mice that were given proteolipid protein with adjuvant to induce EAE. Strikingly, EAE was prevented in a prophylactic treatment regimen of anti-CD40L, and, when EAE was induced in another cohort of animals, CD40L antibody treatment significantly reduced disease severity in an active treatment paradigm [54]. It was later shown that genetic deficiency in CD40L [55] or antibody-mediated blockade of CD40L [56] resulted in attenuation of Th1 differentiation and effector function, including marked inhibition of the Th1 cytokine IFN-γ and reduced numbers of encephalitogenic effector T cells. In an effort to further understand the nature of the CD40-CD40L interaction responsible for promotion of EAE, Becher and colleagues used a bone marrow reconstitution system to determine which CD40-expressing cells were responsible for promoting EAE [57]. In that report, the authors showed that CD40 expression by parenchymal microglia was responsible for recruitment/retention of encephalitogenic T cells in EAE. Strikingly, treatment of microglia with a combination of granulocyte macrophage-colony stimulating factor and CD40L has been shown to promote differentiation of these cells into cells that (1) express the pan-DC marker CD11c, (2) morphologically resemble DCs, and (3) secrete the Th1-promoting cytokine IL-12 p70 [58]. Such CD11c+ CD11b+ "DC-like" microglia were found in EAE brain lesions in inflammatory foci containing T cells, and exhibited potent stimulation of allogeneic T cell proliferation versus CD11c- CD11b+ microglia [58]. Although their origin was not determined, it was recently shown that "CNS DCs" (possibly "DC-like" microglia) are responsible for activation of naïve T cells in response to endogenous myelin epitopes (termed "epitope spreading"), and this process was initiated in the CNS as opposed to the peripheral lymphoid organs [59]. Thus, in the context of EAE, CD40-CD40L interaction on microglia seems to promote adaptive function of these cells, resulting in a "DC-like" activated microglia phenotype that promotes encephalitogenic Th1 cell differentiation and effector function.

## Activation of microglia after CD40 ligation in Alzheimer disease: a shift from innate to adaptive response

### Alzheimer disease and microglial responses to β-amyloid

Alzheimer disease (AD) is the most common dementia and is characterized by insidious onset in late life with progressive decline in memory and other cognitive functions. Definitive diagnosis of AD is made at autopsy, based on the neuropathological hallmarks of extracellular amyloid plaques [largely comprised of β-amyloid (Aβ) peptides, derived from the proteolysis of amyloid precursor protein (APP)] and intracellular neurofibrillary tangles. In addition, brain inflammation, characterized by reactive astrocytes and microglia (but very low levels of infiltrating T cells), is found in close vicinity of amyloid plaques in AD and in transgenic mouse models of the disease (for a review see [60]). It has been suggested that activated microglia play a key role in AD pathogenesis as they secrete pro-inflammatory innate cytokines such as TNF-α and IL-1β, which have been shown to promote neuronal injury at high levels [61,63]. Furthermore, there is a large body of evidence that non-steroidal anti-inflammatory drug (NSAID) use is associated with reduced risk for AD in humans [64-66], (for a review see [67]), and NSAID treatment of AD mice results in reduced amyloid plaque burden concomitant with ameliorated microglial activation [68-70]. Work done in Maxfield's laboratory showed that challenge of microglia with labeled Aβ peptides promotes phagocytosis but poor degradation of soluble or fibrillar Aβ *via *scavenger receptors [71-73]. Using knockout mice, his laboratory showed that the class A scavenger receptor (type I and II) is the predominant scavenger receptor responsible for Aβ uptake by microglia, with other scavenger receptors playing a more minor role (including the class B scavenger receptor CD36) [74].

### Microglial responses to β-amyloid in the context of CD40 ligation

We previously showed that, while murine microglial challenge with soluble Aβ peptides alone does not elicit TNF-α secretion, co-stimulation provided in the form of CD40 ligation (either via CD40L or an agonistic CD40 antibody) results in TNF-α production being synergistically affected [41]. Further, microglia cultured from AD mice deficient in CD40L demonstrate reduced TNF-α secretion versus CD40L-sufficient AD mouse microglia [42]. This form of microglial activation in CD40L-sufficient AD mice is pathogenic, as CD40L-deficient AD mice demonstrate reduced activated (CD11b+) microglia, an effect that is associated with mitigated abnormal hyper-phosphorylation of *tau *protein (a key indicator of neuronal stress) [42]. Furthermore, genetic ablation of CD40L or administration of a CD40L-neutralizing antibody markedly reduces amyloid plaques in mouse models of AD, effects that are associated with mitigated astrocytosis and microgliosis ([75], for review see [76,77]). Recently, overproduction of microglia-associated CD40 and of astrocyte-derived CD40L was found in and around β-amyloid plaques in AD patient brain [78,79], raising the possibility that the CD40-CD40L interaction may contribute to AD pathogenesis by promoting brain inflammation.

In order to better understand the form of microglial activation affected by Aβ plus CD40L stimulation, we examined innate and adaptive activation of murine microglia challenged with Aβ in the presence or absence of CD40L co-stimulation [80]. When microglia were challenged with fluorescent-tagged synthetic human Aβ alone, they mounted a time-dependent phagocytic response (from 15 min to 60 min) which could be enhanced by Fc receptor stimulation using an anti-human Aβ antibody (clone BAM-10). This phagocytic response to Aβ alone was not associated with production of the pro-inflammatory innate cytokines TNF-α, IL-6, or IL-1β, a result similar to that seen when microglia are challenged with apoptotic cells and mount an anti-inflammatory, pro-phagocytic innate response [81]. Importantly, CD40L treatment opposed this phagocytic response, as determined by measuring both cell-associated Aβ and free extracellular Aβ. As mentioned above, Maxfield's laboratory demonstrated that microglia slowly degrade phagocytosed Aβ peptides [71-73]. We examined the ability of microglia to degrade Aβ peptides by first pulsing them with Aβ and then chasing these cells after 1 hour of culture in the presence or absence of CD40L stimulation. Using this experimental approach, we found that CD40L also retarded microglial clearance of the peptide. We further assessed putative modulation of microglial Aβ phagocytosis by cytokines known to promote effector T cell function, and found that the pro-inflammatory Th1-type cytokines IFN-γ and TNF-α inhibited Aβ phagocytosis whereas the anti-inflammatory Th2-type cytokines IL-4 and IL-10 boosted this response.

Having established that CD40 ligation attenuates innate (phagocytic) activation of microglia challenged with Aβ, we then examined the role of CD40 ligation in APC function of Aβ-treated microglia by first determining if Aβ peptides could be co-localized with MHC II. Interestingly, CD40 ligation promoted "loading" of Aβ peptides onto the MHC II molecule as determined by double immunofluorescence microscopy or immunoprecipitation assays. Finally, we determined whether this Aβ-MHC II co-localization was functional by first pre-treating microglia with Aβ in the presence or absence of CD40L, co-culturing these microglia with CD4+ T cells, and then measuring cytokine levels in co-cultured media. Interestingly, Aβ plus CD40L pre-treatment of microglia resulted in markedly enhanced levels of the Th1-promoting cytokines IL-6, TNF-α, IL-2, and IFN-γ. These effects on enhanced cytokine production could be blocked by the addition of an antagonistic CD40 antibody, confirming the requirement of the CD40-CD40L interaction *per se *in this phenomenon. It is interesting that another group found that IFN-γ treatment of microglia promotes APC function of these cells when they are challenged with Aβ [82]. Thus, it seems that when microglia encounter Aβ in the context of co-simulation (e.g., CD40L), their activation phenotype is biased away from innate, phagocytic activation and towards adaptive, APC function.

## Microglial activation in Alzheimer disease immunotherapy: differences between mice and men

In a seminal report, Schenk and colleagues showed that peripheral immunization of the PDAPP mouse model of AD with Aβ_1–42 _peptide resulted in high antibody titers, a small fraction of which (0.1%, [83]) crossed the blood-brain-barrier and entered the brain parenchyma [84]. Most importantly, these authors found that Aβ_1–42 _vaccination markedly diminished β-amyloid plaque burden [84]. These authors also found evidence of cells in the brains of the Aβ_1–42 _immunized animals that contained Aβ. Many of these cells stained for the activated microglia marker MHC II and phenotypically resembled activated microglia, suggesting that these cells were able to phagocytose Aβ deposits. In a follow-up report, Bard and colleagues supported this hypothesis by showing *ex vivo *that certain antibodies against Aβ peptides could trigger microglial phagocytosis and subsequent clearance of Aβ through the Fc receptor [83-85]. Clearance of brain amyloid-β deposits was beneficial, as Aβ_1–42_-vaccinated mice had markedly reduced cognitive impairment as assayed by behavioral testing in AD mice [86,87]. Thus, in mouse models of AD, innate (phagocytic) microglial activation mediated by the Fc receptor in the presence of antibody-opsonized Aβ appears beneficial rather than deleterious.

Based on the above-mentioned data, a human clinical trial was begun to peripherally administer a synthetic Aβ_1–42 _peptide (AN-1792) with an adjuvant to AD patients. Unfortunately, the trial was halted when a small percentage of patients developed aseptic T cell meningoencephalitis. This response most likely occurred because of an immune reaction to Aβ mediated by infiltrating T cells [88]. In the post-mortem brain of one patient who died as a consequence of this side-effect of treatment, there was significant clearance of Aβ plaques in parts of the neocortex and, in other areas where plaques remained, Aβ-immunoreactivity was associated with microglia [89]. It is not yet clear whether this fulminate infiltration of T cells in AD patients who developed aseptic T cell meningoencephalitis was due to adaptive activation of microglia, but this is a distinct possibility given that microglia did seem to recognize antibody-opsonized Aβ [89,90]. These results indicate the potentially damaging and overwhelming effects of a full-blown T cell autoimmune response, which does not normally occur in AD, and which may have been mediated by adaptively activated microglia.

## Conclusion

**Figure 2:**
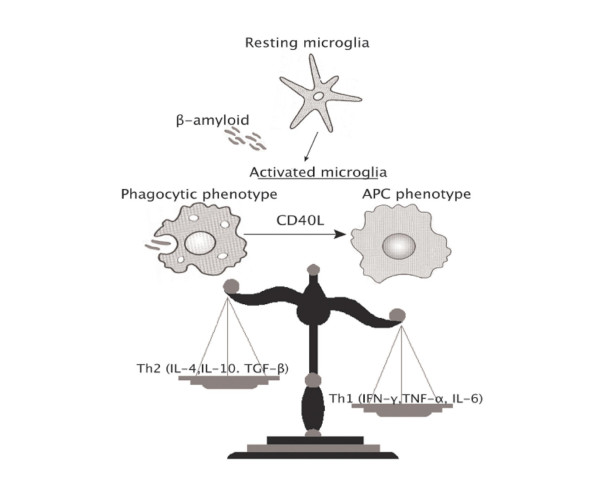
Model for innate versus adaptive microglial activation responses. In the context of β-amyloid challenge, microglia activate a phagocytic response. If co-stimulated with CD40 ligand, a shift from innate activation to adaptive antigen-presenting cell response ensues. Additionally, certain anti-inflammatory Th2-type cytokines shift this balance back towards innate phagocytic response, while some pro-inflammatory Th1-associated cytokines tip the balance further towards adaptive activation of microglia. See the text and Table 1 for references. Abbreviations used: APC, antigen presenting cell; CD40L, CD40 ligand; Th1, CD4+ T helper cell type I response; Th2, Th type II response; TGF, transforming growth factor; IL, interleukin; IFN, interferon, TNF, tumor necrosis factor.

Accumulating evidence has revealed that microglial "activation" is not simply one phenotypic manifestation. Here, we suggest a model wherein microglial cells exist in at least two functionally discernable states once "activated", namely a phagocytic phenotype (innate activation) or an antigen presenting phenotype (adaptive activation), as governed by their stimulatory environment. When challenged with certain PAMPs (particularly CpG-DNA), murine microglia seem to activate a "mixed" response characterized by enhanced phagocytosis and pro-inflammatory cytokine production as well as adaptive activation of T cells. In the EAE model, murine microglia seem to largely support an adaptive activation of encephalitogenic T cells in the presence of the CD40-CD40 ligand interaction. In the context of Aβ challenge, CD40 ligation is able to shift activated microglia from innate to adaptive activation. Further, it seems that the cytokine milieu that microglia are exposed to biases these cells to innate activation (i.e., anti-inflammatory Th2-associated cytokines such as IL-4, IL-10, and perhaps TGF-β1) or an adaptive form of activation (i.e., pro-inflammatory Th1-associated cytokines such as IFN-γ, IL-6, and TNF-α; summarized in Fig. 2). Not all forms of microglial activation are deleterious, as activated microglia may serve a protective role as was shown in Aβ_1–42_-immunized mouse models of AD. It seems that enhanced microglial phagocytosis of β-amyloid plaques is at least partly responsible for the therapeutic benefit in these animals, so perhaps stimulation of innate microglial activation contributes to these reported benefits. In conclusion, if we can learn how to better harness microglia in order to produce specific forms of microglial activation, this could be key in turning a pathogenic cell into a therapeutic modality.

## Competing interests

The author(s) declare that they have no completing interests.

## Authors' contributions

T.T. provided an initial outline of the areas to be covered. V.N. and J.T. wrote the first draft. T.T. performed the experiments described in Fig. 1. V.N. and T.T. edited the references. T.T. and J.T. revised and edited the final manuscript.

## References

[B1] Pessac B, Godin I, Alliot F (2001). [Microglia: origin and development]. Bull Acad Natl Med.

[B2] Alliot F, Godin I, Pessac B (1999). Microglia derive from progenitors, originating from the yolk sac, and which proliferate in the brain. Brain Res Dev Brain Res.

[B3] Eglitis MA, Mezey E (1997). Hematopoietic cells differentiate into both microglia and macroglia in the brains of adult mice. Proc Natl Acad Sci USA.

[B4] Brazelton TR, Rossi FM, Keshet GI, Blau HM (2000). From marrow to brain: expression of neuronal phenotypes in adult mice. Science.

[B5] Mezey E, Chandross KJ, Harta G, Maki RA, McKercher SR (2000). Turning blood into brain: cells bearing neuronal antigens generated in vivo from bone marrow. Science.

[B6] Priller J, Flugel A, Wehner T, Boentert M, Haas CA, Prinz M, Fernandez-Klett F, Prass K, Bechmann I, de Boer BA (2001). Targeting gene-modified hematopoietic cells to the central nervous system: use of green fluorescent protein uncovers microglial engraftment. Nat Med.

[B7] Qureshi ST, Medzhitov R (2003). Toll-like receptors and their role in experimental models of microbial infection. Genes Immun.

[B8] Yamamoto M, Takeda K, Akira S (2004). TIR domain-containing adaptors define the specificity of TLR signaling. Mol Immunol.

[B9] Zhang D, Zhang G, Hayden MS, Greenblatt MB, Bussey C, Flavell RA, Ghosh S (2004). A toll-like receptor that prevents infection by uropathogenic bacteria. Science.

[B10] Tabeta K, Georgel P, Janssen E, Du X, Hoebe K, Crozat K, Mudd S, Shamel L, Sovath S, Goode J (2004). Toll-like receptors 9 and 3 as essential components of innate immune defense against mouse cytomegalovirus infection. Proc Natl Acad Sci USA.

[B11] Janeway CA, Medzhitov R (2002). Innate immune recognition. Annu Rev Immunol.

[B12] Medzhitov R, Janeway C (2000). The Toll receptor family and microbial recognition. Trends Microbiol.

[B13] Medzhitov R, Janeway CA (2000). How does the immune system distinguish self from nonself?. Semin Immunol.

[B14] Medzhitov R, Janeway CA (2002). Decoding the patterns of self and nonself by the innate immune system. Science.

[B15] Iwasaki A, Medzhitov R (2004). Toll-like receptor control of the adaptive immune responses. Nat Immunol.

[B16] Goldsby R, Kindt T, Osborne B, Kuby J, Goldsby R (2002). Mononuclear Phagocytes. Immunology.

[B17] Adler H, Peterhans E, Jungi TW (1994). Generation and functional characterization of bovine bone marrow-derived macrophages. Vet Immunol Immunopathol.

[B18] Blander JM, Medzhitov R (2004). Regulation of phagosome maturation by signals from toll-like receptors. Science.

[B19] Forman HJ, Torres M (2001). Redox signaling in macrophages. Mol Aspects Med.

[B20] Tsukada N, Miyagi K, Matsuda M, Yanagisawa N (1994). Expression of Fc epsilon R2/CD23 and p55 IL-2R/CD25 on peripheral blood macrophages/monocytes in multiple sclerosis. J Neuroimmunol.

[B21] Blom AB, Radstake TR, Holthuysen AE, Sloetjes AW, Pesman GJ, Sweep FG, van de Loo FA, Joosten LA, Barrera P, van Lent PL, van den Berg WB (2003). Increased expression of Fcgamma receptors II and III on macrophages of rheumatoid arthritis patients results in higher production of tumor necrosis factor alpha and matrix metalloproteinase. Arthritis Rheum.

[B22] Gregory CD, Devitt A (2004). The macrophage and the apoptotic cell: an innate immune interaction viewed simplistically?. Immunology.

[B23] Fujiwara N, Kobayashi K (2005). Macrophages in inflammation. Curr Drug Targets Inflamm Allergy.

[B24] Banchereau J, Briere F, Caux C, Davoust J, Lebecque S, Liu YJ, Pulendran B, Palucka K (2000). Immunobiology of dendritic cells. Annu Rev Immunol.

[B25] Shortman K, Liu YJ (2002). Mouse and human dendritic cell subtypes. Nat Rev Immunol.

[B26] Hornung V, Rothenfusser S, Britsch S, Krug A, Jahrsdorfer B, Giese T, Endres S, Hartmann G (2002). Quantitative expression of toll-like receptor 1–10 mRNA in cellular subsets of human peripheral blood mononuclear cells and sensitivity to CpG oligodeoxynucleotides. J Immunol.

[B27] Jarrossay D, Napolitani G, Colonna M, Sallusto F, Lanzavecchia A (2001). Specialization and complementarity in microbial molecule recognition by human myeloid and plasmacytoid dendritic cells. Eur J Immunol.

[B28] Kadowaki N, Ho S, Antonenko S, Malefyt RW, Kastelein RA, Bazan F, Liu YJ (2001). Subsets of human dendritic cell precursors express different toll-like receptors and respond to different microbial antigens. J Exp Med.

[B29] Ito T, Amakawa R, Kaisho T, Hemmi H, Tajima K, Uehira K, Ozaki Y, Tomizawa H, Akira S, Fukuhara S (2002). Interferon-alpha and interleukin-12 are induced differentially by Toll-like receptor 7 ligands in human blood dendritic cell subsets. J Exp Med.

[B30] Hemmi H, Kaisho T, Takeda K, Akira S (2003). The roles of Toll-like receptor 9, MyD88, and DNA-dependent protein kinase catalytic subunit in the effects of two distinct CpG DNAs on dendritic cell subsets. J Immunol.

[B31] Olson JK, Miller SD (2004). Microglia initiate central nervous system innate and adaptive immune responses through multiple TLRs. J Immunol.

[B32] Bsibsi M, Ravid R, Gveric D, van Noort JM (2002). Broad expression of Toll-like receptors in the human central nervous system. J Neuropathol Exp Neurol.

[B33] Kielian T, Mayes P, Kielian M (2002). Characterization of microglial responses to Staphylococcus aureus: effects on cytokine, costimulatory molecule, and Toll-like receptor expression. J Neuroimmunol.

[B34] Kielian T, Esen N, Bearden ED (2005). Toll-like receptor 2 (TLR2) is pivotal for recognition of S. aureus peptidoglycan but not intact bacteria by microglia. Glia.

[B35] Iliev AI, Stringaris AK, Nau R, Neumann H (2004). Neuronal injury mediated via stimulation of microglial toll-like receptor-9 (TLR9). Faseb J.

[B36] Dalpke AH, Schafer MK, Frey M, Zimmermann S, Tebbe J, Weihe E, Heeg K (2002). Immunostimulatory CpG-DNA activates murine microglia. J Immunol.

[B37] Lehnardt S, Lachance C, Patrizi S, Lefebvre S, Follett PL, Jensen FE, Rosenberg PA, Volpe JJ, Vartanian T (2002). The toll-like receptor TLR4 is necessary for lipopolysaccharide-induced oligodendrocyte injury in the CNS. J Neurosci.

[B38] Lehnardt S, Massillon L, Follett P, Jensen FE, Ratan R, Rosenberg PA, Volpe JJ, Vartanian T (2003). Activation of innate immunity in the CNS triggers neurodegeneration through a Toll-like receptor 4-dependent pathway. Proc Natl Acad Sci USA.

[B39] Alexopoulou L, Holt AC, Medzhitov R, Flavell RA (2001). Recognition of double-stranded RNA and activation of NF-kappaB by Toll-like receptor 3. Nature.

[B40] Jack CS, Arbour N, Manusow J, Montgrain V, Blain M, McCrea E, Shapiro A, Antel JP (2005). TLR Signaling Tailors Innate Immune Responses in Human Microglia and Astrocytes. J Immunol.

[B41] Wang T, Town T, Alexopoulou L, Anderson JF, Fikrig E, Flavell RA (2004). Toll-like receptor 3 mediates West Nile virus entry into the brain causing lethal encephalitis. Nat Med.

[B42] Tan J, Town T, Paris D, Mori T, Suo ZM, Crawford F, Mattson MP, Flavell RA, Mullan M (1999). Microglial activation resulting from CD40-CD40L interaction after beta-amyloid stimulation. Science.

[B43] Vogel SN, Fitzgerald KA, Fenton MJ (2003). TLRs: differential adapter utilization by toll-like receptors mediates TLR-specific patterns of gene expression. Mol Interv.

[B44] Hemmi H, Akira S (2005). TLR signalling and the function of dendritic cells. Chem Immunol Allergy.

[B45] Olsson T (1995). Cytokine-producing cells in experimental autoimmune encephalomyelitis and multiple sclerosis. Neurology.

[B46] Swanborg RH (1995). Experimental autoimmune encephalomyelitis in rodents as a model for human demyelinating disease. Clin Immunol Immunopathol.

[B47] Cornet A, Vizler C, Liblau R (1998). [Experimental autoimmune encephalomyelitis]. Rev Neurol (Paris).

[B48] van Kooten C, Banchereau J (2000). CD40-CD40 ligand. J Leukoc Biol.

[B49] Grewal IS, Flavell RA (1998). CD40 and CD154 in cell-mediated immunity. Annu Rev Immunol.

[B50] Nguyen VT, Walker WS, Benveniste EN (1998). Post-transcriptional inhibition of CD40 gene expression in microglia by transforming growth factor-beta. Eur J Immunol.

[B51] Carson MJ, Reilly CR, Sutcliffe JG, Lo D (1998). Mature microglia resemble immature antigen-presenting cells. Glia.

[B52] Havenith CE, Askew D, Walker WS (1998). Mouse resident microglia: isolation and characterization of immunoregulatory properties with naive CD4+ and CD8+ T-cells. Glia.

[B53] Tan J, Town T, Paris D, Placzek A, Parker T, Crawford F, Yu H, Humphrey J, Mullan M (1999). Activation of microglial cells by the CD40 pathway: relevance to multiple sclerosis. Journal of Neuroimmunology.

[B54] Gerritse K, Laman JD, Noelle RJ, Aruffo A, Ledbetter JA, Boersma WJ, Claassen E (1996). CD40-CD40 ligand interactions in experimental allergic encephalomyelitis and multiple sclerosis. Proc Natl Acad Sci USA.

[B55] Grewal IS, Foellmer HG, Grewal KD, Xu J, Hardardottir F, Baron JL, Janeway CA, Flavell RA (1996). Requirement for CD40 ligand in costimulation induction, T cell activation, and experimental allergic encephalomyelitis. Science.

[B56] Howard LM, Miga AJ, Vanderlugt CL, Dal Canto MC, Laman JD, Noelle RJ, Miller SD (1999). Mechanisms of immunotherapeutic intervention by anti-CD40L (CD154) antibody in an animal model of multiple sclerosis. J Clin Invest.

[B57] Becher B, Durell BG, Miga AV, Hickey WF, Noelle RJ (2001). The clinical course of experimental autoimmune encephalomyelitis and inflammation is controlled by the expression of CD40 within the central nervous system. J Exp Med.

[B58] Fischer HG, Reichmann G (2001). Brain dendritic cells and macrophages/microglia in central nervous system inflammation. J Immunol.

[B59] McMahon EJ, Bailey SL, Castenada CV, Waldner H, Miller SD (2005). Epitope spreading initiates in the CNS in two mouse models of multiple sclerosis. Nat Med.

[B60] Akiyama H, Barger S, Barnum S, Bradt B, Bauer J, Cole GM, Cooper NR, Eikelenboom P, Emmerling M, Fiebich BL (2000). Inflammation and Alzheimer's disease. Neurobiol Aging.

[B61] Meda L, Cassatella MA, Szendrei GI, Otvos L, Baron P, Villalba M, Ferrari D, Rossi F (1995). Activation of microglial cells by beta-amyloid protein and interferon-gamma. Nature.

[B62] Barger SW, Harmon AD (1997). Microglial activation by Alzheimer amyloid precursor protein and modulation by apolipoprotein E. Nature.

[B63] McGeer EG, McGeer PL (1998). The importance of inflammatory mechanisms in Alzheimer disease. Exp Gerontol.

[B64] Stewart WF, Kawas C, Corrada M, Metter EJ (1997). Risk of Alzheimer's disease and duration of NSAID use. Neurology.

[B65] in t' Veld BA, Ruitenberg A, Hofman A, Launer LJ, van Duijn CM, Stijnen T, Breteler MM, Stricker BH (2001). Nonsteroidal antiinflammatory drugs and the risk of Alzheimer's disease. N Engl J Med.

[B66] Zandi PP, Anthony JC, Hayden KM, Mehta K, Mayer L, Breitner JC (2002). Reduced incidence of AD with NSAID but not H2 receptor antagonists: the Cache County Study. Neurology.

[B67] Szekely CA, Thorne JE, Zandi PP, Ek M, Messias E, Breitner JC, Goodman SN (2004). Nonsteroidal anti-inflammatory drugs for the prevention of Alzheimer's disease: a systematic review. Neuroepidemiology.

[B68] Lim GP, Yang F, Chu T, Chen P, Beech W, Teter B, Tran T, Ubeda O, Ashe KH, Frautschy SA, Cole GM (2000). Ibuprofen suppresses plaque pathology and inflammation in a mouse model for Alzheimer's disease. Journal of Neuroscience.

[B69] Lim GP, Yang F, Chu T, Gahtan E, Ubeda O, Beech W, Overmier JB, Hsiao-Ashe K, Frautschy SA, Cole GM (2001). Ibuprofen effects on Alzheimer pathology and open field activity in APPsw transgenic mice. Neurobiology of Aging.

[B70] Lim GP, Chu T, Yang FS, Beech W, Frautschy SA, Cole GM (2001). The curry spice curcumin reduces oxidative damage and amyloid pathology in an Alzheimer transgenic mouse. Journal of Neuroscience.

[B71] Paresce DM, Ghosh RN, Maxfield FR (1996). Microglial cells internalize aggregates of the Alzheimer's disease amyloid beta-protein via a scavenger receptor. Neuron.

[B72] Paresce DM, Chung H, Maxfield FR (1997). Slow degradation of aggregates of the Alzheimer's disease amyloid beta-protein by microglial cells. J Biol Chem.

[B73] Brazil MI, Chung H, Maxfield FR (2000). Effects of incorporation of immunoglobulin G and complement component C1q on uptake and degradation of Alzheimer's disease amyloid fibrils by microglia. J Biol Chem.

[B74] Chung H, Brazil MI, Irizarry MC, Hyman BT, Maxfield FR (2001). Uptake of fibrillar beta-amyloid by microglia isolated from MSR-A (type I and type II) knockout mice. Neuroreport.

[B75] Tan J, Town T, Crawford F, Mori T, DelleDonne A, Crescentini R, Obregon D, Flavell RA, Mullan MJ (2002). Role of CD40 ligand in amyloidosis in transgenic Alzheimer's mice. Nat Neurosci.

[B76] Town T, Tan J, Mullan M (2001). CD40 signaling and Alzheimer's disease pathogenesis. Neurochem Int.

[B77] Tan J, Town T, Mullan M (2002). CD40-CD40L interaction in Alzheimer's disease. Curr Opin Pharmacol.

[B78] Togo T, Akiyama H, Kondo H, Ikeda K, Kato M, Iseki E, Kosaka K (2000). Expression of CD40 in the brain of Alzheimer's disease and other neurological diseases. Brain Res.

[B79] Calingasan NY, Erdely HA, Altar CA (2002). Identification of CD40 ligand in Alzheimer's disease and in animal models of Alzheimer's disease and brain injury. Neurobiol Aging.

[B80] Townsend KP, Town T, Mori T, Lue LF, Shytle D, Sanberg PR, Morgan D, Fernandez F, Flavell RA, Tan J (2005). CD40 signaling regulates innate and adaptive activation of microglia in response to amyloid beta-peptide. Eur J Immunol.

[B81] Minghetti L, Ajmone-Cat MA, De Berardinis MA, De Simone R (2005). Microglial activation in chronic neurodegenerative diseases: roles of apoptotic neurons and chronic stimulation. Brain Res Brain Res Rev.

[B82] Monsonego A, Imitola J, Zota V, Oida T, Weiner HL (2003). Microglia-mediated nitric oxide cytotoxicity of T cells following amyloid beta-peptide presentation to Th1 cells. J Immunol.

[B83] Bard F, Cannon C, Barbour R, Burke RL, Games D, Grajeda H, Guido T, Hu K, Huang J, Johnson-Wood K (2000). Peripherally administered antibodies against amyloid beta-peptide enter the central nervous system and reduce pathology in a mouse model of Alzheimer disease. Nat Med.

[B84] Schenk D, Barbour R, Dunn W, Gordon G, Grajeda H, Guido T, Hu K, Huang J, Johnson-Wood K, Khan K (1999). Immunization with amyloid-beta attenuates Alzheimer-disease-like pathology in the PDAPP mouse. Nature.

[B85] Bard F, Barbour R, Cannon C, Carretto R, Fox M, Games D, Guido T, Hoenow K, Hu K, Johnson-Wood K (2003). Epitope and isotype specificities of antibodies to beta-amyloid peptide for protection against Alzheimer's disease-like neuropathology. Proc Natl Acad Sci USA.

[B86] Janus C, Pearson J, McLaurin J, Mathews PM, Jiang Y, Schmidt SD, Chishti MA, Horne P, Heslin D, French J (2000). A beta peptide immunization reduces behavioural impairment and plaques in a model of Alzheimer's disease. Nature.

[B87] Morgan D, Diamond DM, Gottschall PE, Ugen KE, Dickey C, Hardy J, Duff K, Jantzen P, DiCarlo G, Wilcock D (2000). A beta peptide vaccination prevents memory loss in an animal model of Alzheimer's disease. Nature.

[B88] Pfeifer M, Boncristiano S, Bondolfi L, Stalder A, Deller T, Staufenbiel M, Mathews PM, Jucker M (2002). Cerebral hemorrhage after passive anti-Abeta immunotherapy. Science.

[B89] Nicoll JA, Wilkinson D, Holmes C, Steart P, Markham H, Weller RO (2003). Neuropathology of human Alzheimer disease after immunization with amyloid-beta peptide: a case report. Nat Med.

[B90] Monsonego A, Weiner HL (2003). Immunotherapeutic approaches to Alzheimer's disease. Science.

[B91] Albert ML, Pearce SF, Francisco LM, Sauter B, Roy P, Silverstein RL, Bhardwaj N (1998). Immature dendritic cells phagocytose apoptotic cells via alphavbeta5 and CD36, and cross-present antigens to cytotoxic T lymphocytes. J Exp Med.

[B92] Tait JF, Smith C (1999). Phosphatidylserine receptors: role of CD36 in binding of anionic phospholipid vesicles to monocytic cells. J Biol Chem.

[B93] Coraci IS, Husemann J, Berman JW, Hulette C, Dufour JH, Campanella GK, Luster AD, Silverstein SC, El-Khoury JB (2002). CD36, a class B scavenger receptor, is expressed on microglia in Alzheimer's disease brains and can mediate production of reactive oxygen species in response to beta-amyloid fibrils. Am J Pathol.

[B94] Brawand P, Fitzpatrick DR, Greenfield BW, Brasel K, Maliszewski CR, De Smedt T (2002). Murine plasmacytoid pre-dendritic cells generated from Flt3 ligand-supplemented bone marrow cultures are immature APCs. J Immunol.

[B95] Kim WK, Ganea D, Jonakait GM (2002). Inhibition of microglial CD40 expression by pituitary adenylate cyclase-activating polypeptide is mediated by interleukin-10. J Neuroimmunol.

[B96] Prilliman KR, Lemmens EE, Palioungas G, Wolfe TG, Allison JP, Sharpe AH, Schoenberger SP (2002). Cutting edge: a crucial role for B7-CD28 in transmitting T help from APC to CTL. J Immunol.

[B97] Quaranta MG, Tritarelli E, Giordani L, Viora M (2002). HIV-1 Nef induces dendritic cell differentiation: a possible mechanism of uninfected CD4(+) T cell activation. Exp Cell Res.

[B98] Spisek R, Bretaudeau L, Barbieux I, Meflah K, Gregoire M (2001). Standardized generation of fully mature p70 IL-12 secreting monocyte-derived dendritic cells for clinical use. Cancer Immunol Immunother.

[B99] Wyss-Coray T, Lin C, Yan F, Yu GQ, Rohde M, McConlogue L, Masliah E, Mucke L (2001). TGF-beta1 promotes microglial amyloid-beta clearance and reduces plaque burden in transgenic mice. Nat Med.

